# Structural and Functional Interrogation of Selected Biological Nitrogen Removal Systems in the United States, Denmark, and Singapore Using Shotgun Metagenomics

**DOI:** 10.3389/fmicb.2018.02544

**Published:** 2018-10-26

**Authors:** Medini K. Annavajhala, Vikram Kapoor, Jorge Santo-Domingo, Kartik Chandran

**Affiliations:** ^1^Department of Earth and Environmental Engineering, Columbia University, New York, NY, United States; ^2^Department of Civil and Environmental Engineering, University of Texas, San Antonio, TX, United States; ^3^U.S. Environmental Protection Agency, Office of Research and Development, Cincinnati, OH, United States

**Keywords:** BNR, anammox, metagenomics, global survey, deammonification, nitritation-anammox

## Abstract

Conventional biological nitrogen removal (BNR), comprised of nitrification and denitrification, is traditionally employed in wastewater treatment plants (WWTPs) to prevent eutrophication in receiving water bodies. More recently, the combination of selective ammonia to nitrite oxidation (nitritation) and autotrophic anaerobic ammonia oxidation (anammox), collectively termed deammonification, has also emerged as a possible energy- and cost-effective BNR alternative. Herein, we analyzed microbial diversity and functional potential within 13 BNR processes in the United States, Denmark, and Singapore operated with varying reactor configuration, design, and operational parameters. Using next-generation sequencing and metagenomics, gene-coding regions were aligned against a custom protein database expanded to include all published aerobic ammonia oxidizing bacteria (AOB), nitrite oxidizing bacteria (NOB), anaerobic ammonia oxidizing bacteria (AMX), and complete ammonia oxidizing bacteria (CMX). Overall contributions of these N-cycle bacteria to the total functional potential of each reactor was determined, as well as that of several organisms associated with denitrification and/or structural integrity of microbial aggregates (biofilm or granules). The potential for these engineered processes to foster a broad spectrum of microbial catabolic, anabolic, and carbon assimilation transformations was elucidated. Seeded sidestream DEMON® deammonification systems and single-stage nitritation-anammox moving bed biofilm reactors (MBBRs) and a mainstream Cleargreen reactor designed to enrich in AOB and AMX showed lower enrichment in AMX functionality than an enriched two-stage nitritation-anammox MBBR system treating mainstream wastewater. Conventional BNR systems in Singapore and the United States had distinct metagenomes, especially relating to AOB. A hydrocyclone process designed to recycle biomass granules for mainstream BNR contained almost identical structural and functional characteristics in the overflow, underflow, and inflow of mixed liquor (ALT) rather than the expected selective enrichment of specific nitrifying or AMX organisms. Inoculum used to seed a sidestream deammonification process unexpectedly contained <10% of total coding regions assigned to AMX. These results suggest the operating conditions of engineered bioprocesses shape the resident microbial structure and function far more than the bioprocess configuration itself. We also highlight the advantage of a systems- and metagenomics-based interrogation of both the microbial structure and potential function therein over targeting of individual populations or specific genes.

## Introduction

Engineered biological nitrogen removal (BNR) processes employ mixed microbial communities for removing nitrogenous pollutants (ammonia, nitrite, nitrate) from wastewater to prevent eutrophication in receiving water bodies. *Conventionally considered*, influent ammonia is oxidized to nitrite and nitrate by two distinct types of autotrophic nitrifying bacteria (ammonia-oxidizing bacteria, AOB, and nitrite-oxidizing bacteria, NOB, respectively), and nitrate is reduced by chemoorganoheterotrophic denitrifying bacteria to ultimately form dinitrogen gas (N_2_; Lu et al., 2014). However, the excessive energy usage and costs associated with both the aeration input for nitrification and, in some cases, externally added organic carbon for heterotrophic denitrification have led to the exploration of alternative “shortcut” nitrogen removal pathways.

Anaerobic ammonia oxidation, or anammox, represents such a shortcut, and is mediated by anaerobic ammonia-oxidizing bacteria, AMX, which co-convert ammonia and nitrite to N_2_ via hydrazine (N_2_H_2_), an intermediate unique to this process (Strous et al., 2006; Kartal et al., 2011). Through suppression of NOB activity to prevent competition for nitrite as a substrate, partial nitritation (the incomplete oxidation of ammonia to nitrite) by AOB can be combined with anammox in a process referred to as deammonification to significantly reduce required oxygenation and inorganic carbon usage (Ahn et al., 2008). Furthermore, complete ammonia oxidation (comammox) to nitrate in a single organism (CMX) rather than a mixed community of AOB and NOB has been recently characterized (Daims et al., 2015; van Kessel et al., 2015). In a related study, the ubiquity of CMX bacteria in a variety of full-scale wastewater treatment processes has been shown using shotgun metagenomics (Annavajhala et al., 2018).

As the use of novel BNR configurations and strategies becomes increasingly widespread, deeper understanding of the diverse microbial communities employed and their ability to transform nitrogen in wastewater streams must be pursued (Lackner et al., 2014). Molecular techniques such as polymerase chain reaction (PCR) and fluorescence *in-situ* hybridization (FISH) have traditionally been employed to this end, but their scope in the analysis of novel or unknown engineered bioreactor populations and potential or extant function is limited. PCR can only identify or quantify, at most, several targeted genes, and requires prior knowledge of these gene sequences in relevant organisms in order to design and optimize primer sets. Similarly, FISH requires the use of hybridization probes which must also be designed based on a previous understanding of target sequences.

Next-generation sequencing (NGS), on the other hand, permits much deeper systems-interrogation capabilities and possible reconciliation between the identities and concentrations of microbial protagonists with potential and extant metabolic function in a given system. NGS also allows for massive throughput of sequence collection and characterization, without the need for prior selection and detailed knowledge regarding microbial protagonists, genes or pathways of interest. However, despite the ability of NGS to provide large amounts of information about environmental microbial communities, some limitations must be recognized. Firstly, the relative lack of well-curated reference genomes for organisms involved in the systems being studied, for instance, the conventional and shortcut BNR processes herein, poses tangible obstacles in our analysis. For example, to date only several full or draft metagenomes of AMX species have been published (Gori et al., 2011; Speth et al., 2012, 2015; van de Vossenberg et al., 2013; Ali et al., 2015, 2016; Oshiki et al., 2015; Park et al., 2017), resulting in a relatively narrow range of structural and functional genomic templates to compare our results with. Secondly, metagenomics-based interrogation of biological systems does not reveal measures of extant function and activity, which might be crucial and still need to be probed toward overall engineered system characterization. In this study, we aim to develop a systems-level understanding of the characteristics and capabilities of multiple field-scale BNR reactors. Ultimately, the insights provided by surveys such as this can foster discussion as to how increased understanding of microbial communities and their functional potential can inform best practices for implementation and provide metrics to measure success of enhanced and shortcut BNR.

**Figure 1:**
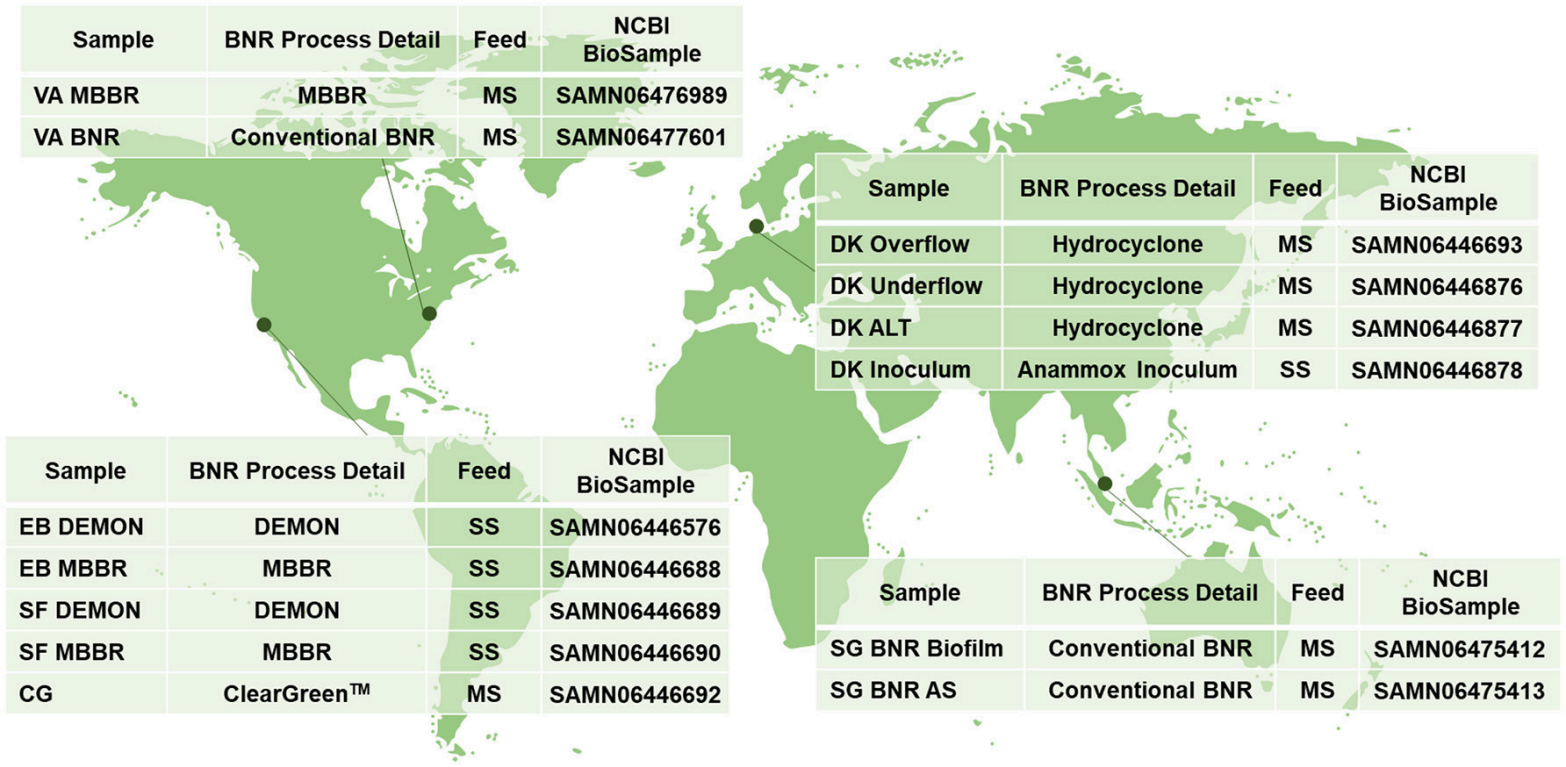
Sampling locations and characteristics; MBBR, Moving Bed Biofilm Reactor; BNR, Biological Nitrogen Removal; MS, Mainstream Wastewater; DEMON, DEamMONification; SS, Sidestream Wastewater; Cleargreen, Cyclic Low Energy Ammonium Removal; AS, Activated Sludge.

Accordingly, next-generation sequencing techniques were applied to samples from six global BNR wastewater treatment plants (WWTPs) employing varying reactor configurations (Figure 1). The use of metagenomics allowed for the characterization of the microbial ecology of these systems, as well as the comparison of the metabolic (functional) *potential* of each reactor, specifically with a focus here on nitrogen catabolism and carbon anabolism. In addition, possible links between structure-function of the microbial communities and the varied reactor configurations in which they were fostered, along with influent characteristics, were explored.

## Materials and methods

### Reactor sampling

Biomass was collected from WWTPs in the United States, Singapore, and Denmark (Figure 1). Granules from DEMON® deammonification systems (EB DEMON, SF DEMON) and biofilm from single-stage sidestream deammonification moving bed biofilm reactors (MBBR; EB MBBR, SF MBBR) were collected, along with biomass from a Cleargreen pilot reactor (CG) and biofilm from a two-stage mainstream nitritation-anammox MBBR system (VA MBBR). Activated sludge and opportunistically enriched biomass from conventional BNR processes (VA BNR, SG BNR AS, SG BNR Biofilm) were also collected. Mixed liquor from a mainstream BNR process (DK ALT) was analyzed along with the overflow and underflow from an attached hydrocyclone designed for retention of dense biomass granules (DK Overflow, DK Underflow). Lastly, a commercial AMX inoculum stream used to seed a sidestream nitritation-anammox reactor (DK Inoculum) was included in this study. Biomass was pelleted and shipped to Columbia University on dry ice from each respective location, and stored at −80°C.

### Next-generation sequencing and bioinformatics

DNA was extracted manually from the 13 biomass samples using the MoBio PowerLyzer PowerSoil DNA Isolation Kit (Qiagen, CA) and quantified using dsDNA HS assay on the Qubit 2.0 Fluorimeter (Life Technologies, NY), with input DNA levels as recommended by the protocol. Whole-genome (shotgun) libraries were prepared using the TruSeq DNA PCR-Free HT Library Preparation Kit (Illumina, CA) and sequenced using an Illumina MiSeq sequencer at the Cincinnati Children's Hospital DNA Core Facility (Illumina, CA) with pair-ended kits targeting 250 bp fragment length. Resulting pair-ended reads were merged and screened with mothur ver. 1.36.1 to remove ambiguous bases, cap homopolymeric regions at 10 bp, and select merged pair-ended reads between 250 and 450 bp (Table 1; Schloss et al., 2009). Filtered merged reads from each sample were then aligned against a custom database of proteins which expanded the currently available NCBI non-redundant (nr) protein database to include genes from two recently published AMX metagenomes [*Ca*. “Scalindua profunda” (van de Vossenberg et al., 2013) and *Ca*. “B. caroliniensis” (Park et al., 2017)] and three sequenced comammox-capable organisms (CMX) [*Ca*. “Nitrospira inopinata” (Daims et al., 2015), *Ca*. “Nitrospira nitrificans,” and *Ca*. “Nitrospira nitrosa”(van Kessel et al., 2015)], as well as protein sequences from draft metagenome-derived genomes published more recently (Speth et al., 2016; Lawson et al., 2017). NCBI's BLASTX program with a maximum e-value of 1e-20 and up to 10 hits per read (Acland et al., 2014). Alignments were then manually curated through additional filtering by percentage identity (≥90; Table 1). In addition, taxonomy and functional importance were assigned to each aligned read; we required consensus regarding both protein alignment and genus of taxonomic assignment between the top five hits for an affirmative classification. Read counts were normalized using the reads per kilobase mapped (RPKM) method to account for potential read count biases caused by differences in gene length or total library size. AOB (genera: *Nitrosomonas, Nitrosospira, Nitrosococcus*), NOB (genera: *Nitrobacter, Nitrospira*), AMX (genera: *Candidatus* “Brocadia,” *Candidatus* “Jettenia,” *Candidatus* “Kuenenia,” *Candidatus* “Scalindua”), and some heterotrophic organisms capable of denitrification (genera: *Chlorobium, Ignavibacterium, Chloroflexi*) were used to focus the study on microbial transformations of nitrogen in wastewater (Supplementary Table 1). Community profiles and pathway-specific functional heat maps were generated using the “heatmaps2” and “ggplots2” packages in R (ver. 3.3.0; R Core Team, 2017).

**Table 1:** Sequencing output, quality control, and alignment statistics.

	**Assembly**	**Quality control**		**Alignment**	
**Sample**	**Merged paired-ended reads^a^ (million)**	**Filtered merged reads (million)**	**Ave. filtered merged reads length (bp)**	**Filtered merged reads aligned to complete custom database (%)**	**Filtered merged reads aligned to N-cycle organisms^b^ (%)**
EB DEMON	4.54	3.39	394	74.91	16.62
EB MBBR	4.15	3.15	400	79.20	15.17
SF DEMON	4.32	3.28	398	92.09	18.51
SF MBBR	4.19	3.16	396	78.87	18.36
CG	4.92	3.63	395	80.71	12.05
VA MBBR	6.54	4.52	388	73.13	18.07
SG biofilm	5.75	4.20	391	66.89	8.19
SG AS	4.09	2.92	393	81.16	8.02
VA BNR	5.11	3.86	394	72.71	9.65
DK overflow	4.74	3.44	390	90.54	9.33
DK underflow	4.41	3.33	400	87.47	8.84
DK ALT	7.26	5.05	387	84.22	8.39
DK inoculum	5.36	3.99	394	87.20	19.74

a *Referred to as contigs in the mothur pipeline documentation (see Schloss et al., 2009)*.

b *See Supplementary Table 1*.

## Results and discussion

The 13 samples produced an average of 3.69 million quality filtered, merged pair-ended reads, at an average length of 394 bp (Table 1). Between 66.9 and 92.1% of filtered merged reads aligned to the custom-expanded BLASTX database, representing the total coding DNA sequences (CDS) in each metagenome, and 8.02–19.74% of filtered merged reads aligned specifically to coding regions produced by N-transforming microorganisms selected for in-depth analysis (Table 1 and Supplementary Table 1). The 13 samples were grouped into four categories by process design for analysis and discussion. The first category included **nitritation-anammox** systems, as represented by the DEMON, MBBR, and Cleargreen systems (denoted by EB DEMON, EB MBBR, SF DEMON, SF MBBR, VA MBBR, and CG). The second category included **conventional BNR** processes (denoted by SG BNR Biofilm, SG BNR AS, VA BNR). The third category was the **hydrocyclone based mainstream BNR process**, from which overflow, underflow, and mixed liquor (ALT) samples were characterized (denoted by DK Overflow, Underflow, ALT). The fourth category was an **AMX inoculum biomass** sample which was used to seed a sidestream deammonification reactor (denoted by DK Inoculum). The nitritation-anammox, conventional BNR, and hydrocyclone processes were all operationally stable and not experiencing system failure near time of sampling. Therefore, no major disruptions to previously observed or stoichiometrically calculated microbial community structure and function, as outlined below, were expected.

### Nitritation-anammox processes

Of the six nitritation-anammox processes studied, four (EB DEMON, EB MBBR, SF DEMON, SF MBBR) were designed to treat sidestream wastewater (with an influent total Kjeldahl nitrogen (TKN) of 600–1,200 mg-N/L, T = 25–40°C), while the other two (CG, VA MBBR) were designed for mainstream wastewater treatment (influent TKN ~60 mg N/L and ambient temperature).

#### Sidestream nitritation-anammox processes

**Figure 2:**
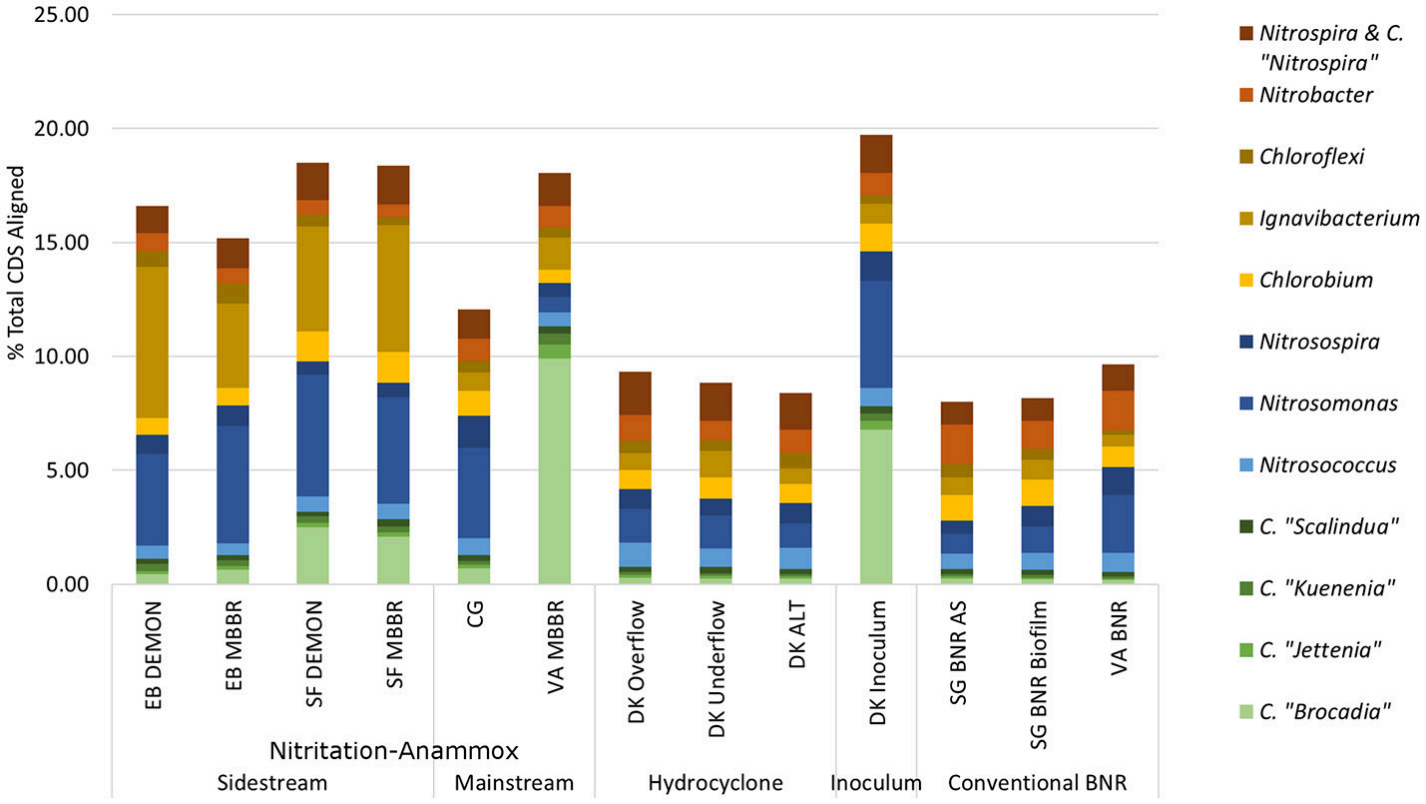
Distribution of metagenome coding regions amongst key N-transforming organisms. Remainder of % total CDS aligned assigned to non-N-cycling organisms. *CDS, coding DNA sequences; CMX, complete ammonia-oxidizing bacteria*.

Metagenomic community profiles in the sidestream nitritation-anammox systems, EB DEMON and MBBR and SF DEMON and MBBR, were distinct despite geographically close sampling sites (Figures 1, 2). DEMON® systems aim for the enrichment and recycling of desired deammonification microorganisms (AOB, AMX) and out-selection of the undesired NOB by promoting biomass granulation through sequencing batch reactor (SBR) operation and pH control (Wett et al., 2015). Previous work has shown that the enriched granules provide an ideal anaerobic internal environment for AMX growth and activity, allowing AMX to out-compete NOB for nitrite as a substrate and achieve effective deammonification (Vlaeminck et al., 2010; Winkler et al., 2011). The MBBR biofilm, on the other hand, is grown on carriers which are distributed through the entire reactor volume for maximal substrate contact. These biofilm structures foster substrate (including nitrogen, carbon, and oxygen) gradients, which should allow AOB to thrive on the oxygen- and ammonia-exposed outer layers, while AMX thrive in the deeper anaerobic layers using ammonia and AOB-produced nitrite as their substrates (Mehrdad et al., 2014).

*C*. “Brocadia” and *C*. “Kuenenia” have been previously reported as major AMX genera found in wastewater systems depending on seed characteristics (Park et al., 2010a). Similarly*, Nitrosomonas* spp.-related AOB have previously been reported as a majority AOB group in wastewater systems, although the lower influent TKN concentrations in the mainstream systems (CG, VA MBBR) were expected to lead to increased relative contributions of *Nitrosospira* spp. related AOB (Ahn et al., 2008; Norton et al., 2008; Vázquez-Padín et al., 2010; Kim et al., 2011; Ye et al., 2012). At the EB plant, both DEMON and MBBR systems revealed unexpectedly limited AMX contribution to the total metagenome (1.11 and 1.27% of total CDS assigned to AMX, respectively), with the majority of AMX reads assigned to *Candidatus* “Brocadia”-related spp. (39.3 and 49.22% of total AMX CDS; Figure 2 and Supplementary Table 2). In the SF DEMON and MBBR reactors, AMX-assigned reads accounted for a higher percentage of the metagenome (3.18 and 2.84%), again mostly assigned to *C*. “Brocadia” (78.5 and 72.9%), yet also contributed <5% of total CDS.

AOB, on the other hand, accounted for 5.46 and 6.58% of total CDS in the EB DEMON and MBBR reactors (73.9 and 78.4% of AOB CDS assigned to *Nitrosomonas*) and 6.61 and 6.02% of total CDS in the SF DEMON and MBBR reactors (80.8 and 77.6% of AOB assigned to *Nitrosomonas* spp.).

NOB contributed 2.01 and 1.99% of total CDS in the EB DEMON and MBBR metagenomes. The detected NOB were dominated by *Nitrospira*-related spp. (60.7 and 66.0% *Nitrospira-*related spp., respectively), and 2.31 and 2.23% of CDS in SF DEMON and MBBR metagenomes (72.3% and 75.4% *Nitrospira-*related spp., respectively). Overall, the abundance of NOB was higher than expected, especially in the four sidestream processes (EB DEMON and MBBR, SF DEMON and MBBR), which were expected to harbor lower overall NOB coding region abundance and functional potential, due to inhibition by high levels of both free ammonia and free nitrous acid, as well as limiting dissolved oxygen (DO) concentrations (Ahn et al., 2011). Further, under the limiting DO concentrations and gradients typical of DEMON and MBBR systems, *Nitrospira* spp.-related NOB were expected to be more abundant than the more oxygen-sensitive *Nitrobacter* spp.-related NOB (Huang et al., 2010). On the other hand, although *Nitrobacter* spp. have been observed in sidestream partial nitrification processes (Ahn et al., 2008, 2011), *Nitrospira* spp. were found to be dominant in the biofilm but not suspension of a sidestream biofilm-suspended process (Park et al., 2015). Therefore, our results align well with DO-based but not for ${\text{NO}}_{2}^{-}$-N concentration-based selection of NOB detected in these systems.

*Ignavibacterium album* is a filamentous *Chlorobi-*lineage bacterium capable of denitrification which has been previously associated with AMX growth in biofilms (Liu et al., 2012). In all four sidestream nitritation-anammox metagenomes, coding regions assigned to heterotrophic *Ignavibacterium*-related spp. were even more abundant than those for the principal N-cycle bacteria (6.62 and 3.68% in EB DEMON and MBBR, and 4.61 and 5.57% in SF DEMON and MBBR), indicating the capacity for chemoorganoheterotrophic denitrification as well as deammonification within these structures. Thus, while the expected genera constituted the majority of each metagenome's functional potential, the overall contribution of desired AOB and AMX organisms was low (<10% of total CDS) in all four reactors.

**Figure 3:**
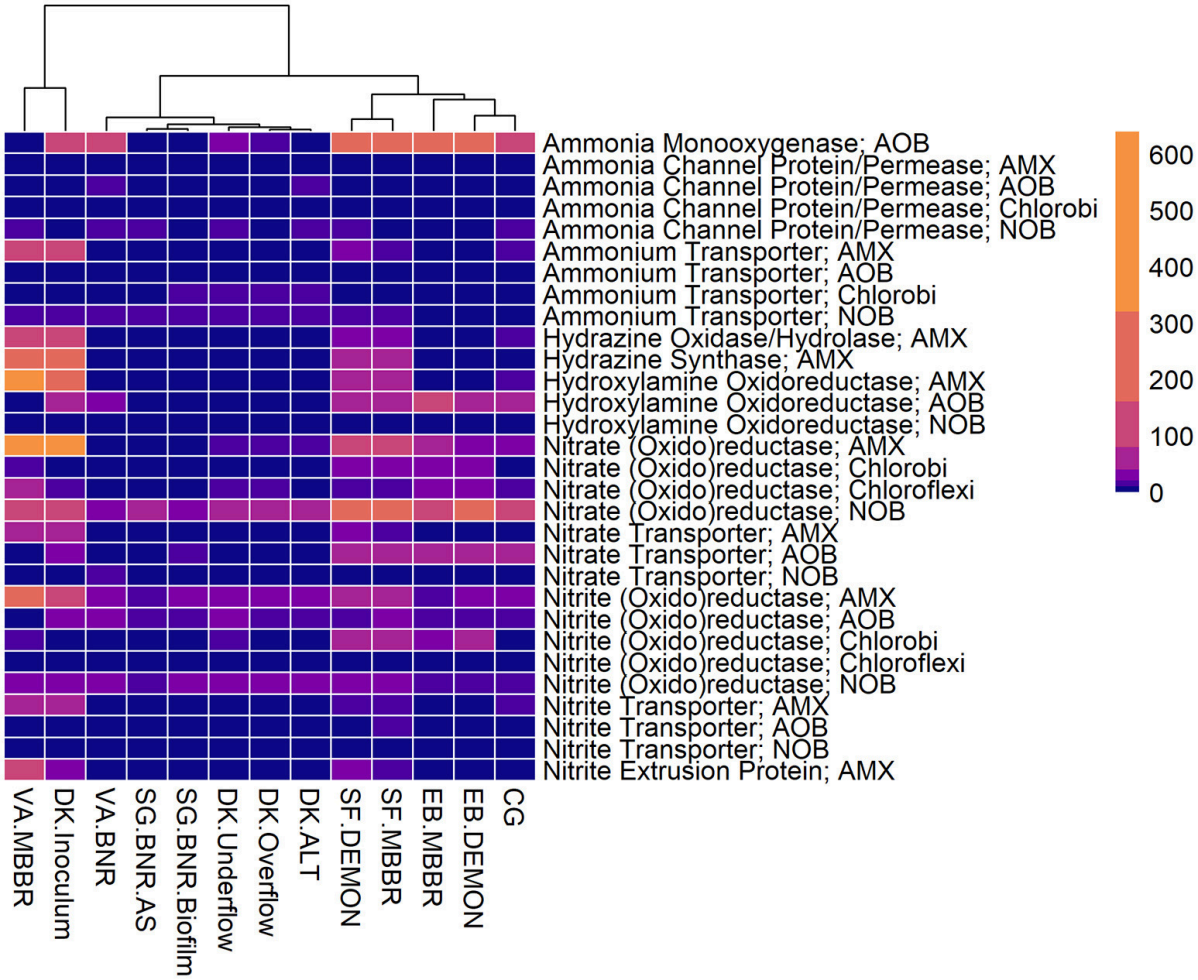
Relative contributions of key microorganisms to potential production of proteins involved in nitrogen metabolism. Unsupervised clustering of samples shows close clustering of the MBBR process from VA (VA.MBBR) and AMX inoculum (DK.Inoculum), conventional BNR processes (SG.BNR.AS, SG.BNR.Biofilm, VA.BNR), hydrocyclone (DK.Underflow, DK.Overflow, DK.ALT), and nitritation-anammox (SF.DEMON, SF.MBBR, EB.MBBR, EB.DEMON, CG) samples. AOB, ammonia-oxidizing bacteria; AMX, anaerobic ammonia-oxidizing bacteria; NOB, nitrite-oxidizing bacteria; RPKM, reads per kilobase per million mapped.

**Figure 4:**
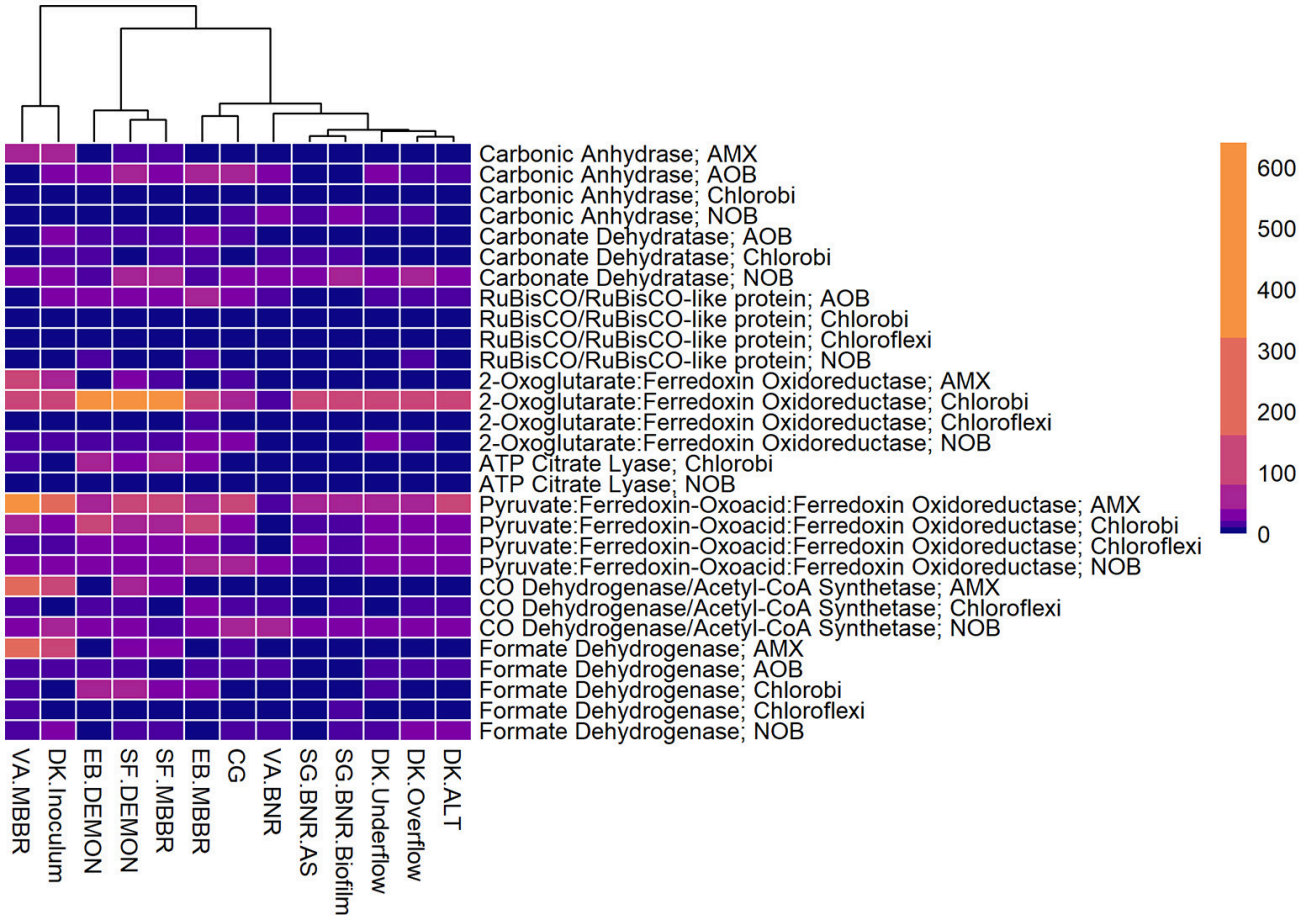
Relative contributions of key microorganisms to potential production of proteins involved in carbon fixation. Unsupervised clustering of samples shows close clustering of the MBBR process from VA (VA.MBBR) and AMX inoculum (DK.Inoculum), conventional BNR processes (SG.BNR.AS, SG.BNR.Biofilm, VA.BNR), hydrocyclone (DK.Underflow, DK.Overflow, DK.ALT), and nitritation-anammox (SF.DEMON, SF.MBBR, EB.MBBR, EB.DEMON, CG) samples. AOB, ammonia-oxidizing bacteria; AMX, anaerobic ammonia-oxidizing bacteria; NOB, nitrite-oxidizing bacteria; RPKM, reads per kilobase per million mapped.

All four sidestream nitritation-anammox processes studied (EB DEMON and MBBR, SF DEMON and MBBR) contained coding regions for ammonia monooxygenase, the key enzyme involved in the conversion of ammonia to hydroxylamine, and hydroxylamine oxidoreductases, required for the conversion of hydroxylamine to nitrite (Figure 3, Supplementary Figure 1, and Supplementary Table 3). Despite low overall contribution to the metagenome (RPKM ≤ 75), the key functional enzymes of AMX (hydrazine synthase, hydrazine oxidase/hydrolase) were also present in the metagenome, along with genes involved in carbon fixation for biosynthesis by AMX through the Wood-Ljungdahl Pathway (CO dehydrogenase/acetyl-coA synthetase and formate dehydrogenase; Figure 4, Supplementary Figure 2, and Supplementary Table 4) reflecting the fingerprint of anammox function. Genes encoding for heterotrophic denitrification by *Chloroflexi*- and *Chlorobi*-related nitrate reductases were also prevalent (Figure 3 and Supplementary Table 3). In addition, *Chlorobi* and *Chloroflexi*-assigned regions contributed significantly to the potential of these four systems to produce the three rate-limiting enzymes involved in the reverse tricarboxylic acid (rTCA) cycle: 2-oxoglutarate:ferrodoxin oxidoreductase, ATP citrate lyase, and pyruvate:ferrodoxin oxidoreductase (Figure 4 and Supplementary Table 4; Campbell and Cary, 2004). In all four sidestream nitritation-anammox processes, the genomic inventory of nitrite oxidoreductase from NOB and AMX was comparable, and NOB-related carbon fixation genes involved in the rTCA cycle were present, indicating incomplete out-selection of NOB and potential dissipation of savings in energy and organic carbon through NOB-mediated nitrate oxidation (Figures 2, 3; Ahn et al., 2008). While the overall functional potential and community profiles of these sidestream nitritation-anammox processes varied between the two WWTPs, EB, and SF, reactor configuration *(*i.e., DEMON vs. MBBR) did not impact the relative contributions of AOB, NOB, or AMX to overall reactor functional potential (Figures 2, 3). This suggests that the treatment plant-specific characteristics and operational conditions played a larger role than process design in determining the microbial community structure and functional capabilities of these systems.

#### Mainstream nitritation-anammox processes

The patented Cyclic Low Energy Ammonia Removal (Cleargreen) process (denoted herein as CG) was, like the DEMON® systems, operated in a single-stage partial nitritation-anammox-based deammonification mode, in a pilot SBR reactor (Degremont Infilco, 2015). The CG process uses a series of cycles to enrich the reactor biomass in AOB and AMX. While initially designed for treating sidestream wastewater post-anaerobic digestion, here the CG process was used to treat mainstream wastewater.

The single-stage mainstream nitritation-anammox CG pilot system contained 1.27% of total coding regions assigned to AMX (54.6% *C*. “Brocadia”; Figure 2 and Supplementary Table 2). This represents a smaller proportion of total CDS than in the reactors at SF (SF DEMON, SF MBBR), as expected due to sidestream treatment conditions at SF, which should conceptually enrich a higher fraction of AMX. Notably, the percentage of AMX CDS was higher in the CG reactor than in the sidestream nitritation-anammox reactors at EB (EB DEMON, EB MBBR), despite treating mainstream wastewater. AOB accounted for 6.13% of total CDS (64.7% of AOB CDS assigned to *Nitrosomonas*), and NOB CDS were identified (2.25% of total CDS). Of these, 43.8% of NOB CDS were assigned to *Nitrobacter* spp.-related NOB and 56.2% of NOB CDS were assigned to *Nitrospira* spp.-related NOB. Unlike the DEMON and MBBR processes, the Cleargreen pilot reactor was operated with multiple feeding cycles of aerated phases followed by anoxic phases, and therefore was not expected to completely out-select *Nitrobacter* spp.-related NOB (Degremont Infilco, 2015). The contribution of *Ignavibacterium spp*. to the CG metagenome (0.79% of total CDS) was lower than in the four sidestream systems, again likely due to the aerobic phases within each feeding cycle in the Cleargreen reactor. Corresponding to anammox metabolism, the CG reactor community showed the genomic signature for hydrazine synthase and hydrazine oxidase/hydrolase (Figure 3 and Supplementary Table 3). However, the CG metagenome contained fewer RPKM aligned to these genes compared to the SF sidestream nitritation-anammox metagenomes and higher RPKM for these genes compared to the EB metagenomes, paralleling the relative levels of total AMX CDS (Figures 2, 3, and Supplementary Tables 2, 3). The ineffective out-selection of NOB in the CG reactor was indicated by significant RPKM for nitrite and nitrate oxidoreductase-producing genes attributed to NOB, indicating the potential for NOB-mediated conversion of nitrite to nitrate (Figure 3 and Supplementary Figure 1). Insufficient NOB washout in the CG reactor was also underscored by the presence of NOB-mediated CO_2_-fixation pathways for biosynthesis through RuBisCO (in *Nitrobacter*) and RuBisCO-like protein and the rTCA cycle (*Nitrospira*; Figure 4 and Supplementary Table 4). However, coding regions for AOB RuBisCO genes and AMX genes involved in the Wood-Ljungdahl pathway for carbon fixation had higher RPKM than the NOB CO_2_-fixation pathways, indicating greater potential for AOB and AMX growth as desired from a process engineering perspective (Figure 4 and Supplementary Figure 2).

The pilot-scale mainstream VA MBBR process was comprised of two-stages; an aerated nitritation stage followed by an unaerated deammonification MBBR stage. In the biofilm samples from the VA mainstream anaerobic MBBR reactor (VA MBBR; Figure 1), AMX CDS contributed significantly to the overall biofilm metagenome (11.3% of total CDS), and 87.6% of the AMX CDS were assigned to *C*. “Brocadia” (Figure 2 and Supplementary Table 2). The high proportions of AMX CDS in this mainstream MBBR were surprising, especially when compared to the lower metagenomic contributions of AMX in the four sidestream nitritation-anammox systems (EB DEMON and MBBR, SF DEMON and MBBR). These results indicate that the use of two-stage MBBR processes for nitritation-anammox under mainstream conditions can lead to substantial AMX enrichment, making mainstream anammox viable. Unlike the other nitritation-anammox systems, this mainstream reactor contained comparable levels of *Nitrosomonas* and *Nitrosospira* CDS (out of 1.90% total CDS assigned to AOB, 36.4% *Nitrosomonas* and 31.4% *Nitrosospira*), due to the ability of *Nitrosospira* to better compete with *Nitrosomonas* at low process nitrogen concentrations (Figure 2; Gao et al., 2013).

Some NOB remained in the VA MBBR as seen in the other reactors (2.40% total; 61.7% *Nitrospira* and 38.4% *Nitrobacter*). Low process nitrogen concentration and correspondingly low free nitrous acid concentrations in the mainstream VA MBBR process likely reduced the degree of inhibition of NOB compared to that in the sidestream systems (Ahn et al., 2011). However, the low total genomic inventory of NOB compared to AMX in this case was still promising in terms of NOB out-selection. The likelihood of significant anammox contribution to N-conversion was further supported by the dominance of AMX-related coding regions involved in nitrogen metabolism (RPKM 238 and 236 for hydrazine synthase and hydroxylamine oxidoreductase, compared to RPKM ≤ 75 in all other nitritation-anammox systems; Figure 3 and Supplementary Table 3). The potential of AMX for carbon fixation through rTCA cycle and the Wood-Ljungdahl pathways, as well as the anammox-mediated formation of carbonate by carbonic anhydrase, was also higher in this process compared to even the sidestream processes, in particular both the EB DEMON and MBBR systems (Figures 3, 4 and Supplementary Tables 3, 4).

In sum, these results indicated that although each of these six nitritation-anammox reactors were capable of fostering key anammox and AOB structure and function, overall metagenomic contributions of desired AMX and AOB remained low in the majority of systems, as has been previously shown in full-scale conventional wastewater treatment reactors (Kim et al., 2011; Gao et al., 2013). Interestingly, the mainstream two-stage nitritation-anammox MBBR system (VA MBBR) showed a much more robust potential for anammox than even the sidestream single-stage DEMON and MBBR systems. Additionally, the enrichment of AMX under mainstream conditions can be favored when operated in a two-stage sequential nitritation-anammox mode. However, NOB were not completely out-selected in any of the six mainstream and sidestream energy-efficient BNR systems, and heterotrophic denitrification continued to be a likely possibility, revealing opportunities for improved nitritation-anammox process optimization through community enrichment and monitoring strategies. Importantly, the similarities of the sidestream DEMON and MBBR processes at both EB and SF treatment plants, and the high level of AMX enrichment at the mainstream VA MBBR pilot system, suggest a major role for the process operations and enrichment strategies employed in determining the ultimate functional capabilities of an engineered bioprocess. Furthermore, these results show that appropriate process engineering and operation can make mainstream deammonification a feasible opportunity for extensive energy- and cost-savings in full-scale WWTPs.

### Conventional mainstream BNR systems

The conventional mainstream BNR systems in Singapore and VA contained distinct community and functional profiles. In Singapore, biomass from mixed liquor in an aerated basin (SG BNR AS) as well as biofilm found growing in an anoxic basin (SG BNR Biofilm) were analyzed, while the VA samples was from the aerated zone of a BNR reactor (VA BNR). AOB and NOB, required for full nitrification of ammonia to nitrate, were expected to be present in significant levels, in addition to denitrifiers converting nitrate to nitrogen gas (Supplementary Figure 1). Specifically, *Nitrosomonas* spp.- and *Nitrosospira* spp.-related AOB were expected to dominate each metagenome typical of mainstream wastewater (Kim et al., 2011). Also, *Nitrospira* were expected to compete here as major NOB groups relative to *Nitrobacter* spp., which are more prevalent in sidestream systems (Blackburne et al., 2008; Kouba et al., 2014). The SG biofilm sample (SG BNR Biofilm) was analyzed in order to determine potential AMX presence and functionality, as aggregation in biofilms is typical of AMX-capable organisms due to secretion of extracellular polymeric substances (EPS; Tan et al., 2014). Therefore, some AMX coding regions were expected in the SG BNR Biofilm metagenome. Additionally, filamentous *Ignavibacterium* were expected to be involved in the formation of biofilm structures, and thus contribute more to the biofilm metagenome compared to the two mixed liquor BNR metagenomes.

As expected, low levels of AMX CDS were observed in the two activated sludge metagenomes (0.69% of total CDS assigned to AMX in SG BNR AS, and 0.55% of total CDS in VA BNR; Figure 2 and Supplementary Table 2). AOB were twice as prevalent in VA BNR than in SG BNR AS (2.12% of total CDS in SG BNR AS, and 4.59% in VA BNR), and were assigned mainly to *Nitrosomonas* spp. with some *Nitrosospira* spp. in both metagenomes as expected. Levels of NOB, on the other hand, were comparable in these two samples (2.73% NOB CDS in SG BNR AS and 2.89% NOB in VA BNR). As expected in mainstream nitrification, *Nitrobacter* were present at higher levels than Nitrospira spp. in these samples (63.2% *Nitrobacter* and 36.9% *Nitrospira* in SG BNR AS, and 60.1% *Nitrobacter* and 39.9% *Nitrospira* in VA BNR). Despite the expectation of opportunistic AMX growth in process biofilms, a low degree of AMX biomass enrichment was found in the SG BNR Biofilm (0.69% of total CDS). From a functional standpoint, the key enzymes coding sequences responsible for anammox activity were not found at significant levels (RPKM < 0.005) in either of these conventional BNR systems, making opportunistic anammox unlikely (Figure 3 and Supplementary Table 3). In both activated sludge samples, low levels of inorganic carbon processing and carbon fixation genes coding for carbonic anhydrase and CO dehydrogenase/acetyl-CoA synthetase related to AMX (Figure 4 and Supplementary Table 4), further underscore low AMX potential in the conventional BNR processes sampled. Also, Ignavibacterium only accounted for 0.87% of total CDS in the SG BNR Biofilm metagenome, compared to 0.75 and 0.53% in the SG BNR AS and VA BNR metagenomes, respectively (Figure 2 and Supplementary Table 2). The similarity of the biofilm and activated sludge metagenomes from SG implied that the biofilm was likely a result of aggregation of the same community found in the suspended activated sludge rather than an enrichment of distinct niche ecology. This is in contrast to a recent example at another municipal WWTP from the United States, where opportunistically growing biofilm was discovered to be AMX (*C*. “B. caroliniensis”; Park et al., 2017).

The VA BNR metagenome contained significant genomic inventory for AOB-specific AMO and HAO (RPKM of 84 and 21, respectively), as well as NOB-specific nitrite oxidoreductase (RPKM of 28), thereby representing the full traditional or canonical nitrification pathway (Figure 3 and Supplementary Figure 1). However, as with the overall community profiles, the SG BNR AS and Biofilm metagenomes contained much fewer coding regions assigned to AOB-mediated ammonia and hydroxylamine oxidation (RPKM of 0.19 and 0.56 in SG BNR AS; 0.89 and 0.99 in the SG Biofilm) compared to the VA BNR metagenome. However, the genomic potential for NOB-specific nitrite oxidoreductase was comparable across the three samples and two processes.

Although these results were in line with expectations, metagenomics allowed for deeper inferences of dominant bacterial community structure and functional capability of AOB and NOB due to our assessment of both nitrogen and carbon cycling pathways.

### Mainstream RAS hydrocyclone process

The hydrocyclone process operated in Denmark was designed to separate high density granules from mainstream return activated sludge (RAS) to be recycled for biomass retention within the mainstream BNR process. Ultimately, AMX retention in the mainstream reactor would be desired once transitioned from a BNR process to a mainstream anammox system to reduce overall treatment costs and energy and chemical usage (Nielsen et al., 2015). The goal of this system was to effectively select for biomass in the underflow with improved settling capability, characteristic of denser granules. The hydrocyclone influent, mixed liquor from the mainstream system (DK ALT), was expected to contain significant levels of AOB, NOB, and denitrifier coding regions needed for conventional BNR. The granules separated into the underflow (DK Underflow) were presumed to contain internally anaerobic areas where organisms such as AMX and heterotrophic denitrifiers could thrive, and outer surface areas well suited for AOB activity (Winkler et al., 2011; Bagchi et al., 2016). In the overflow (DK Overflow), planktonic cells and biomass with decreased settleability was expected (Shi et al., 2016).

Interestingly, the microbial diversity and functional potential in the mainstream mixed liquor (hydrocyclone influent, DK ALT) showed the same profiles as both the overflow and underflow of the hydrocyclone (DK Overflow and Underflow). The DK ALT metagenome contained 2.91% AOB CDS, while the DK Overflow contained 3.41% AOB and the DK Underflow contained 2.98% AOB, with 36.5, 43.6, and 48.1% of AOB CDS assigned to *Nitrosomonas*-related spp. (Figure 2 and Supplementary Table 2); these levels are also comparable to the conventional BNR system from Singapore (SG BNR AS and Biofilm). In the DK ALT, 2.63% of total CDS were assigned to NOB, with 39.2\3% assigned to *Nitrobacter*-related spp. and 60.7% assigned to *Nitrospira*-related spp. The DK Overflow and Underflow similarly contain comparable levels of AOB and NOB (3.03% total NOB, 37.0% *Nitrobacter* and 63.0% *Nitrospira* in the DK Overflow; 2.50% total NOB, 33.9% *Nitrobacter* and 66.1% *Nitrospira* in the DK Underflow). AMX CDS contributed only 0.66% of total CDS in the DK ALT, and only 0.76 and 0.77% of total CDS in both the DK Overflow and Underflow. Surprisingly, the hydrocyclone did not select for *Ignavibacterium*, associated with filament-mediated aggregation of biomass, in the underflow despite its design to enrich in denser, more settleable biomass (Liu et al., 2012). These microbial community profiles indicate no significant separation of microorganism groups between the hydrocyclone overflow and underflow, despite its design to enrich granular biomass in the underflow.

The functional potential of AOB and NOB, in terms of coding regions assigned to ammonia and hydroxylamine oxidation and nitrite oxidation, were comparable across all three samples (Figure 3 and Supplementary Table 3). Also, the potential of the DK ALT, DK Overflow, and DK Underflow samples to produce hydrazine synthesis and oxidation enzymes as well as carbon fixation enzymes in the Wood-Ljungdahl pathway by AMX was minimal compared to metabolic and anabolic pathways in AOB and NOB (Figures 3, 4 and Supplementary Tables 3, 4). Expected differences in the overflow and underflow metagenomes in terms of nitrification enzyme coding regions were not observed (Figure 3 and Supplementary Table 3). Additionally, the levels of coding regions related to carbon fixation, and by extension biomass growth capacity, was unchanged between these samples: the underflow did not contain significantly more coding regions assigned to either the Calvin Bassham Benson (CBB) cycle used by AOB or the Wood-Ljungdahl Pathway used by AMX (Figure 4 and Supplementary Table 4; Chain et al., 2003; de Almeida et al., 2011).

The lack of differentiation amongst the three samples suggest that hydrocyclones designed to separate by biomass density and settleability must be further optimized to achieve the desired community enrichment. Further, these results also show that enrichment of granular biomass in the hydrocyclone underflow does not necessarily lead to enrichment in AOB- and AMX-rich granules. The application of hydrocyclone systems designed to enrich AOB and AMX for nitritation-anammox systems, for example, cannot rely on separation based solely on relative biomass density or settling properties of desired (for e.g., AOB and AMX) vs. undesired (for e.g., NOB) organisms.

### Commercial sidestream AMX biomass

The inoculum for the sidestream nitritation-anammox DEMON process (DK Inoculum) was obtained from a sidestream deammonification process in the Netherlands and used to seed the DEMON reactor in Denmark. This sample was expected to contain significant levels of coding regions assigned to AMX, and significant coding potential for enzymes in the anammox and Wood-Ljungdahl carbon assimilation pathways. Indeed, in the DK Inoculum, 7.80% of total CDS were assigned to AMX, 87.1% of which were assigned specifically to *C*. “Brocadia”-related spp. (Figure 2 and Supplementary Table 2). AOB also contributed significantly to the inoculum metagenome (6.81% total CDS assigned to AOB, 69.2% assigned to *Nitrosomonas-*related spp.). Some NOB (2.65% of total CDS) remained, indicating incomplete washout in the parent anammox process. The metagenomic analysis did indicate enrichment of AMX within the DK Inoculum, although, unexpectedly, the nitritation-anammox system from VA (VA MBBR) still contained greater proportions of AMX coding regions despite treating mainstream wastewater (Figure 2). The DK inoculum, similarly to the other nitritation-anammox samples, contained AMX CDS primarily identified as *Ca*. “Brocadia” spp. The DK Inoculum metagenome contained significant levels of AMO and HAO CDS, as well as HZS and HZO CDS, indicating potential for the desired nitritation and anammox pathways (Figure 3 and Supplementary Table 3). Additionally, anabolic pathways required for growth in both AOB and AMX were represented in this metagenome (Figure 4 and Supplementary Table 4). In general, the inoculum sample was enriched in the AOB and AMX desired for nitritation-anammox. Nevertheless, coding regions assigned to each of these groups remained below 10% of the total functional potential. These findings bring into question the efficacy of using commercial inocula to “seed” BNR processes. Potentially, a better approach could be to enrich native anammox bacteria more suited to any given combination of wastewater characteristics or process operating conditions, as shown previously (Park et al., 2010a).

## Conclusions

Our metagenomics approach revealed diverse microbial community structure and functional potential profiles in a variety of reactor configurations from engineered processes in three countries. We characterized the relative contributions of AMX, AOB, NOB, and other N-cycle bacteria in each reactor to the overall metagenome and the potential to produce specific nitrogen- and carbon-cycling enzymes. Generally, we found higher AMX coding potential in the nitritation-anammox compared to conventional BNR systems as expected. Additionally, the anticipated consortium of AOB, NOB, and/or AMX was broadly identified in each system. However, the two-stage MBBR (VA MBBR) contained a significantly higher proportion of coding regions assigned to AMX and potential to produce key anammox enzymes than other deammonification processes. Also, conventional BNR systems from the United States and Singapore displayed distinct metagenomic profiles. The hydrocyclone designed to retain biomass granules for BNR showed surprisingly similar metagenomic fingerprints in the overflow and underflow, as well as the inflow ALT. This indicated that density-based separation at this plant had not yet resulted in selective enrichment of AOB, NOB, or AMX. Lastly, even an inoculum stream used to seed a sidestream deammonification process was not highly enriched (<10% of coding regions assigned) in AMX.

At the same time, these results raise interesting questions as to the efficacy of overall process design or reactor selection to promote the growth of consistent microbial communities. Traditional principles guiding design of engineered biosystems consider both reactor operational design and cultivation or seeding of the microbial community equally influential on the ultimate community structure and function. Here, our results suggest a critical role for process operating conditions on the resultant biological makeup of each engineered process, perhaps more than the design configuration of the reactor itself. Of note, the percentage of CDS not aligned to organisms traditionally linked to nitrogen cycling provides a rich dataset for potential future studies aimed at either identifying organisms indirectly involved in BNR or organisms with symbiotic or competitive relationships with AOB, NOB, and/or AMX.

Regardless, effectiveness of current methods of reactor startup and biomass retention in anammox systems must be assessed at the metagenomic level at least periodically or during process startup to unveil correspondence between expected and observed reactor performance. Additionally, NGS-based interrogation should be complemented with periodic measurement of microbial community structure using alternative techniques (e.g., FISH, qPCR or other techniques) to understand a system's capabilities. Furthermore, the use of relative abundance does not provide detail on absolute levels of biomass in each reactor, which may be affected by reactor design or operation. One important aspect not revealed by metagenomics pertains to the metabolic activity of the microbial protagonists, which could be obtained through whole-cell or molecular assays such as mRNA measurements (Chandran and Love, 2008; Park et al., 2010b, 2015; Lu et al., 2011). It is also expected that the rapidly expanding database of reference genomes will greatly assist in the interpretation of upcoming NGS based studies. More broadly, however, this study provides a blueprint for the use of metagenomics to assess, quantify, and compare the functional potential of mixed microbial communities, in order to ultimately improve efficacy and efficiency of engineered bioprocesses such as those employed in engineered BNR processes.

## Author contributions

KC and MA designed the study. MA performed metagenomics analyses to develop structural and functional profiles, wrote the initial manuscript draft, and generated all tables and figures. VK processed biomass samples for sequencing. JS-D and KC reviewed the manuscript prior to submission.

### Conflict of interest statement

The authors declare that the research was conducted in the absence of any commercial or financial relationships that could be construed as a potential conflict of interest. The reviewer MA and handling Editor declared their shared affiliation.

## Supplementary material

Supplementary Figure 1 Relevant nitrogen cycling pathways. Nrt, nitrate transporter; nxr/narGH, nitrite oxidoreductases; narK, nitrite/nitrate antiporter; foc/nirC, formate and nitrite transporter; nirS/nirK, nitrite reductase (cytochrome cd1-/iron-containing); nor, nitric oxide reductase; nos, nitrous oxide reductase; hdh/hzo, hydrazine dehydrogenase/hydrazine oxidoreductase; hzs, hydrazine synthase; Nrf/nirB, dissimilatory nitrite reduction to ammonia (DNRA); amo, ammonia monooxygenase; hao, hydroxylamine oxidoreductase; amt, amp, acp, ammonium transporters.

Supplementary Figure 2 Relevant carbon fixation pathways. cah, carbonic anhydrase; cdh, carbonic dehydratase; fdh, formate dehydrogenase; COdh, carbon monoxide dehydrogenase; acs, acetyl-CoA synthetase; cbbLS, RuBisCO; rlp, RuBisCO-like protein; acl, ATP citrate lyase; kgs, α-ketoglutarate synthase; frd, fumarate reductase.

Supplementary Table 1 BNR-capable organisms selected for targeted analysis.

Supplementary Table 2 Percent total aligned coding DNA sequences (CDS) in each biological nitrogen removal (BNR) process aligned to major genera involved in or related to BNR.

Supplementary Table 3 RPKM values indicating relative contributions of key microorganisms to potential production of enzymes involved in nitrogen metabolism.

Supplementary Table 4 RPKM values indicating relative contributions of key microorganisms to potential production of enzymes involved in carbon metabolism.
