# Supplementary figures and images for: Structural and Functional Interrogation of Selected Biological Nitrogen Removal Systems in the United States, Denmark, and Singapore Using Shotgun Metagenomics
Source: Front Microbiol. 2018 Oct 26;9:2544. doi: 10.3389/fmicb.2018.02544 (PMC6212598; doi:10.3389/fmicb.2018.02544)

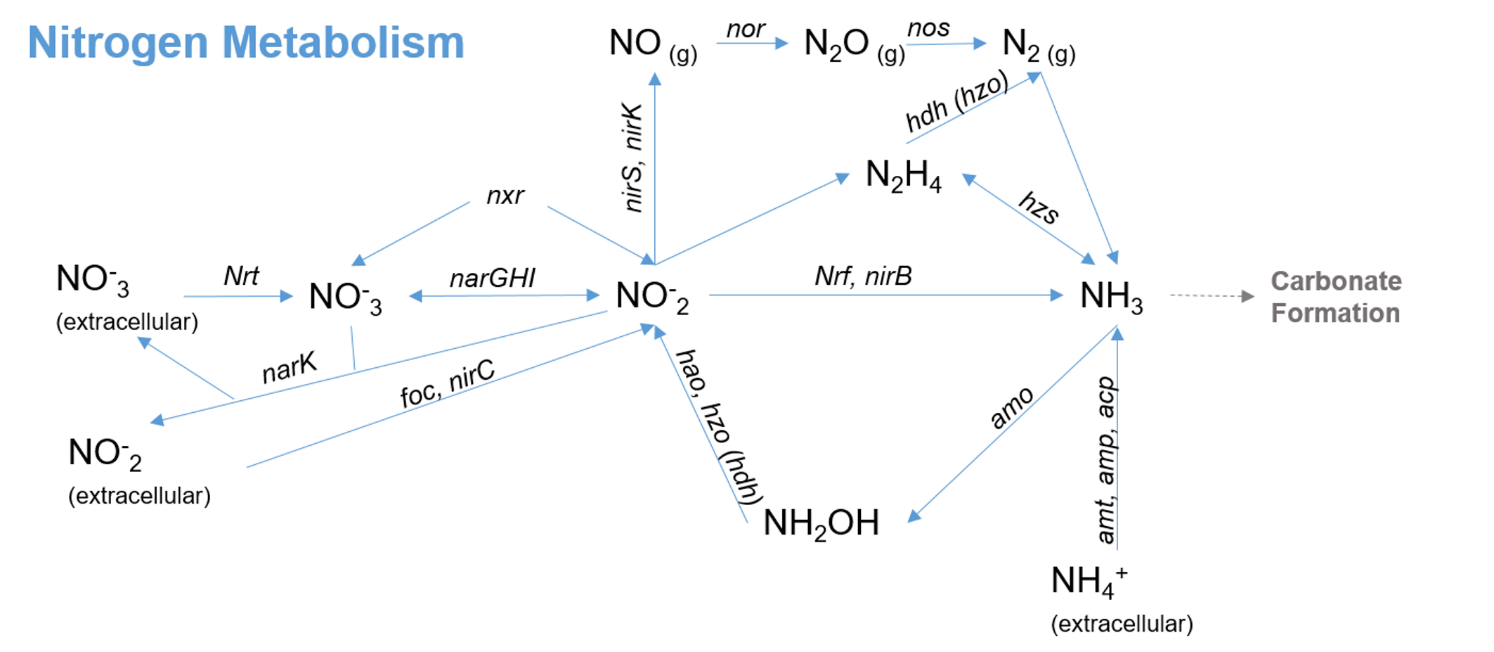

Supplement: Supplementary Figure 1 — Relevant nitrogen cycling pathways. Nrt, nitrate transporter; nxr/narGH, nitrite oxidoreductases; narK, nitrite/nitrate antiporter; foc/nirC, formate and nitrite transporter; nirS/nirK, nitrite reductase (cytochrome cd1-/iron-containing); nor, nitric oxide reductase; nos, nitrous oxide reductase; hdh/hzo, hydrazine dehydrogenase/hydrazine oxidoreductase; hzs, hydrazine synthase; Nrf/nirB, dissimilatory nitrite reduction to ammonia (DNRA); amo, ammonia monooxygenase; hao, hydroxylamine oxidoreductase; amt, amp, acp, ammonium transporters. [file Image_1.tif]

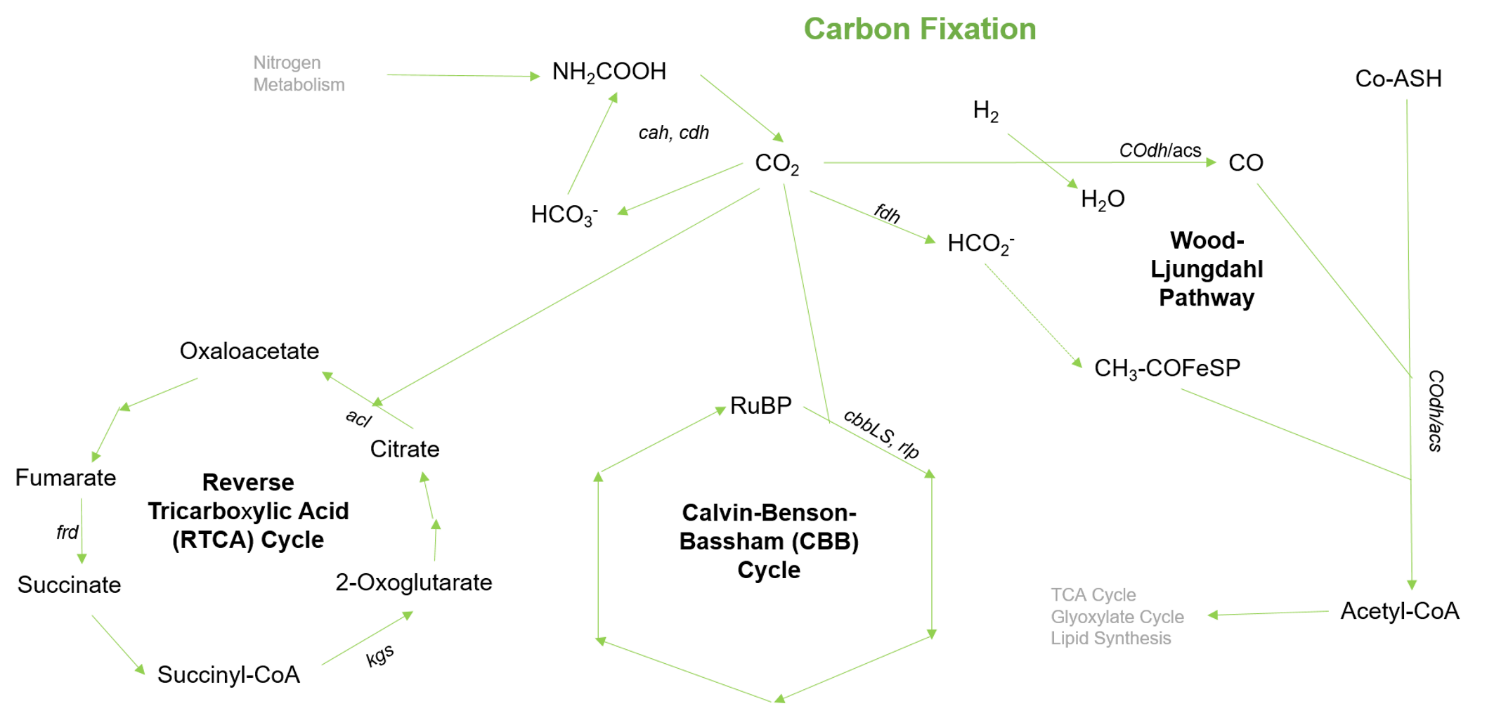

Supplement: Supplementary Figure 2 — Relevant carbon fixation pathways. cah, carbonic anhydrase; cdh, carbonic dehydratase; fdh, formate dehydrogenase; COdh, carbon monoxide dehydrogenase; acs, acetyl-CoA synthetase; cbbLS, RuBisCO; rlp, RuBisCO-like protein; acl, ATP citrate lyase; kgs, α-ketoglutarate synthase; frd, fumarate reductase. [file Image_2.tif]
