# Supplementary material for: Structural and Functional Interrogation of Selected Biological Nitrogen Removal Systems in the United States, Denmark, and Singapore Using Shotgun Metagenomics
Source: Front Microbiol. 2018 Oct 26;9:2544. doi: 10.3389/fmicb.2018.02544 (PMC6212598; doi:10.3389/fmicb.2018.02544)
Supplement: Supplementary Table 1 — BNR-capable organisms selected for targeted analysis. [file Table_1.docx]

Supplementary Table 1. BNR-capable organisms selected for targeted analysis

| AOB ^1^ | NOB ^2^ | AMX ^3^ | Chlorobi | Chloroflexi |
| --- | --- | --- | --- | --- |
| *Nitrosomonas communis* | *Nitrobacter hamburgensis* | *Ca.* “Brocadia caroliniensis” ^4^ | *Chlorobi bacterium* NICIL-2 | *Chloroflexi bacterium* CSP1-4 |
| *Nitrosomonas cryotolerans* | *Nitrobacter sp.* Nb-311A | *Ca.* “Brocadia fulgida” | *Chlorobium chromatii* | *Chloroflexi bacterium* OLB13 |
| *Nitrosomonas eutropha* | *Nitrobacter winogradskyi* | *Ca.* “Brocadia sinica” | *Chlorobium ferroxidans* | *Chloroflexi bacterium* OLB14 |
| *Nitrosomonas sp.* AL212 | *Nitrospira bacterium* SG8_3 | *Ca.* “Kuenenia stuttgartiensis” | *Chlorobium limicola* | *Chloroflexi bacterium* OLB15 |
| *Nitrosomonas sp.* Is79A3 | *Nitrospira bacterium* SG8_35_1 | *Ca.* “Jettenia caeni” | *Chlorobium phaeobacteroides* |  |
| *Nitrosomonas ureae* | *Nitrospira bacterium* SG8_35_4 | *Ca.* “Scalindua brodae” | *Chlorobium phaeovibrioides* |  |
| *Nitrosospira lacus* | *Nitrospira bacterium* SM23_35 | *Ca. “*Scalindua profunda” ^4^ | *Chlorobium* *sp.* GBChlB |  |
| *Nitrosospira multiformis* | *Nitrospira defluvii* |  | *Ignavibacterium album* |  |
| *Nitrosospira sp.* NpAV | *Nitrospira moscoviensis* |  | *Melioribacter roseus* |  |
| *Nitrosococcus halophilus* | *Nitrospira* *sp*. OLB3 |  |  |  |
| *Nitrosococcus oceani* | *Ca. “*Nitrospira nitrosa” ^4,5^ |  |  |  |
| *Nitrosococcus watsonii* | *Ca.* “Nitrospira nitrificans” ^4,5^ |  |  |  |
| *Nitrosomonas europaea* | *Ca. “*Nitrospira inopinata” ^4,5^ |  |  |  |

^1^ AOB: (aerobic) ammonia oxidizing bacteria

^2^ NOB: nitrite oxidizing bacteria

^3^ AMX: anaerobic ammonia oxidizing bacteria

^4^ Manually added to custom expanded *nr* database

^5^ Complete ammonia oxidizers (CMX)
