# Supplementary material for: Structural and Functional Interrogation of Selected Biological Nitrogen Removal Systems in the United States, Denmark, and Singapore Using Shotgun Metagenomics
Source: Front Microbiol. 2018 Oct 26;9:2544. doi: 10.3389/fmicb.2018.02544 (PMC6212598; doi:10.3389/fmicb.2018.02544)
Supplement: Supplementary Table 2 — Percent total aligned coding DNA sequences (CDS) in each biological nitrogen removal (BNR) process aligned to major genera involved in or related to BNR. [file Table_2.docx]

Supplementary Table 2. Percent total aligned coding DNA sequences (CDS) in each biological nitrogen removal (BNR) process aligned to major genera involved in or related to BNR

| **Genus** | **% Total CDS Aligned** | | | | | | | | | | | | |  |
| --- | --- | --- | --- | --- | --- | --- | --- | --- | --- | --- | --- | --- | --- | --- |
|  | EB DEMON | EB MBBR | SF DEMON | SF MBBR | PDR | DK Overflow | DK Underflow | DK ALT | DK Inoculum | SG BNR AS | SG BNR Biofilm | VA MBBR | VA BNR | |
| C. "Brocadia" | 0.43 | 0.62 | 2.50 | 2.07 | 0.69 | 9.91 | 0.29 | 0.26 | 0.26 | 6.79 | 0.27 | 0.22 | 0.20 | |
| C. "Jettenia" | 0.15 | 0.17 | 0.19 | 0.20 | 0.16 | 0.62 | 0.12 | 0.11 | 0.10 | 0.39 | 0.10 | 0.08 | 0.07 | |
| C. "Kuenenia" | 0.31 | 0.26 | 0.30 | 0.27 | 0.17 | 0.47 | 0.12 | 0.12 | 0.10 | 0.31 | 0.09 | 0.12 | 0.08 | |
| C. "Scalindua" | 0.20 | 0.21 | 0.19 | 0.30 | 0.25 | 0.31 | 0.23 | 0.28 | 0.20 | 0.31 | 0.23 | 0.22 | 0.20 | |
| Nitrosococcus | 0.59 | 0.54 | 0.69 | 0.68 | 0.75 | 0.61 | 1.06 | 0.80 | 0.94 | 0.82 | 0.66 | 0.73 | 0.84 | |
| Nitrosomonas | 4.03 | 5.15 | 5.34 | 4.67 | 3.96 | 0.69 | 1.48 | 1.44 | 1.06 | 4.71 | 0.88 | 1.18 | 2.55 | |
| Nitrosospira | 0.84 | 0.89 | 0.58 | 0.67 | 1.42 | 0.60 | 0.86 | 0.75 | 0.90 | 1.28 | 0.57 | 0.89 | 1.21 | |
| Chlorobium | 0.73 | 0.79 | 1.30 | 1.35 | 1.11 | 0.60 | 0.86 | 0.95 | 0.84 | 1.21 | 1.13 | 1.15 | 0.90 | |
| Ignavibacterium | 6.62 | 3.68 | 4.61 | 5.57 | 0.79 | 1.41 | 0.73 | 1.16 | 0.68 | 0.87 | 0.75 | 0.87 | 0.53 | |
| Chloroflexi | 0.69 | 0.88 | 0.51 | 0.36 | 0.50 | 0.44 | 0.54 | 0.48 | 0.68 | 0.39 | 0.60 | 0.50 | 0.20 | |
| Nitrobacter | 0.79 | 0.67 | 0.64 | 0.55 | 0.99 | 0.92 | 1.12 | 0.85 | 1.03 | 0.96 | 1.73 | 1.21 | 1.73 | |
| Nitrospira & C. "Nitrospira" | 1.22 | 1.31 | 1.67 | 1.68 | 1.27 | 1.48 | 1.91 | 1.65 | 1.59 | 1.69 | 1.01 | 1.02 | 1.15 | |
| Other | 83.38 | 84.83 | 81.49 | 81.64 | 87.95 | 81.93 | 90.67 | 91.16 | 91.61 | 80.26 | 91.98 | 91.81 | 90.35 | |
